# Effects of density dependence in a temperate forest in northeastern China

**DOI:** 10.1038/srep32844

**Published:** 2016-09-08

**Authors:** Jie Yao, Xinna Zhang, Chunyu Zhang, Xiuhai Zhao, Klaus von Gadow

**Affiliations:** 1 Key Laboratory for Forest Resources & Ecosystem Processes of Beijing, Beijing Forestry University, No. 35 Qinghua East Road, Haidian District, Beijing 100083, China; 2 Faculty of Forestry and Forest Ecology, Georg-August-University Göttingen, Büsgenweg 5, D-37077 Göttingen, Germany; 3 Department of Forest and Wood Science, University of Stellenbosch, South Africa

## Abstract

Negative density dependence may cause reduced clustering among individuals of the same species, and evidence is accumulating that conspecific density-dependent self-thinning is an important mechanism regulating the spatial structure of plant populations. This study evaluates that specific density dependence in three very large observational studies representing three successional stages in a temperate forest in northeastern China. The methods include standard spatial point pattern analysis and a heterogeneous Poisson process as the null model to eliminate the effects of habitat heterogeneity. The results show that most of the species exhibit conspecific density-dependent self-thinning. In the early successional stage 11 of the 16 species, in the intermediate successional stage 18 of the 21 species and in the old growth stage all 21 species exhibited density dependence after removing the effects of habitat heterogeneity. The prevalence of density dependence thus varies among the three successional stages and exhibits an increase with increasing successional stage. The proportion of species showing density dependence varied depending on whether habitat heterogeneity was removed or not. Furthermore, the strength of density dependence is closely related with species abundance. Abundant species with high conspecific aggregation tend to exhibit greater density dependence than rare species.

A major challenge in ecology is to explain which ecological mechanisms govern the coexistence of species and thus the biodiversity in species-rich communities^1^,^2^. Density-dependent conspecific survival and niche partitioning have been widely discussed. According to the Janzen-Connell Hypothesis (JCH)^3^,^4^, host-specific natural enemies, such as seed predators, herbivores and pathogens, reduce offspring recruitment and survival when surrounded by a high density of conspecific neighbors^3^,^4^, thus regulating population dynamics and facilitating species coexistence in diverse tree communities^1^. This effect, known as Conspecific Negative Density Dependence (CNDD)^5^ is a major mechanism for shaping plant communities from temperate to tropical forests^6^,^7^,^8^,^9^,^10^ and may cause reduced spatial aggregation among individuals of the same species.

A number of studies have provided evidence for the role of that specific density dependence in regulating plant populations in tropical^7^,^11^,^12^,^13^,^14^ and subtropical forests^9^,^15^,^16^,^17^. For example, Peters^11^ found that more than 80% of species tested showed density-dependent mortality at each site of two species-rich tropical forests. Comita and Hubbell^13^ found that the survival of 45 (76.3%) of 59 seedling species was affected by neighborhood density in a 50-ha plot in Panama. Guo *et al*.^17^ found that 66 (75%) of 88 tree species exhibit conspecific density dependence in a species-rich subtropical forest.

Still, the prevalence of conspecific negative density dependence and the life stages which are affected by this dependence remain controversial. Even at similar subtropical regions, inconsistent results have been obtained in different forests. For example, Zhu *et al*.^9^ found that 83.0% of the tested tree species at later life-stages (dbh ≥ 1 cm) showed conspecific negative density dependence in dynamic plots of the Gutianshan forest (Gutianshan FDP). On the other hand, Luo *et al*.^18^ found five out of six (i.e., 83%) tested seedling species at early life-stages (dbh < 1 cm) but only one of these tree species at later life-stages (dbh ≥ 1 cm) showing negative density dependence in the Baishanzu mountains, located in roughly the same subtropical region as the Gutianshan plots in eastern China. Furthermore, there have been only few studies of negative density dependence in temperate forests, in contrast to the numerous studies in tropical and subtropical forests.

A direct way of detecting density dependence is to compare performance. Performance may refer to growth or mortality of individual focal plants in response to increasing density within a nearby conspecific neighborhood^1^. At early life-stages (e.g., the seedling stage) conspecific negative density dependence can be detected by direct observation, due to the susceptibility to the effects of biotic and abiotic neighborhoods resulting in high mortality rates^10^. However, for bigger trees with slower rates of growth and mortality with potential lifespans of several hundreds of years, a few years of observations are too short to detect direct effects of density dependence^19^,^20^. Furthermore, long-term observational data are generally rarely available.

Another approach is to infer density dependence indirectly by comparing the spatial patterns at different life-stages^2^,^9^,^10^,^17^,^19^,^21^,^22^,^23^. Conspecific aggregations could be expected at the earlier life stages due to limited seed dispersal and limited competition with other species^24^. Conspecific negative density dependence may be expected when the degree of conspecific aggregation declines with increasing tree size due to enhanced resource competition, pathogenic effects and allelopathy^1^,^3^,^25^. It seems that few attempts have been made to detect effects of density dependence by comparing changes in the spatial patterns at different life-history stages in temperate forests.

Although the method of comparing spatial patterns is relatively easy to implement and theoretically sound, it is possible that large-scale habitat heterogeneity or plant-plant interactions may generate similar spatial patterns^24^. For example, a species will tend to perform better and show an aggregated spatial distribution in its preferred habitat, regardless of existing negative density-dependence^10^,^26^,^27^. This may result in a positive conspecific density dependence. Large-scale habitat heterogeneity may thus mask the true effects of a negative density dependence. Consequently, it is essential to separate the effects of habitat heterogeneity from the effects of density-dependent self-thinning when evaluating density dependence.

Species composition and diversity, as well as community structures may vary with successional stages^28^,^29^. A large number of individuals and species may immigrate at the early successional stages, but negative density effects and competition for limited resources become more intense over time^29^. As a result, the community loses more and more species and the overall density of individual trees declines. Consequently, an interesting question arises: does the prevalence of negative density dependence in regulating plant populations change along a successional gradient? In this study, we are evaluating the effect of negative density dependence by using point pattern analysis at three successional stages in very large observational studies with mapped trees in a temperate forest of northeastern China.

We explore the prevalence of density dependence after controlling the potentially confounding effects of large-scale habitat heterogeneity by addressing the following specific questions: (1) Is there evidence for negative conspecific density dependence after controlling for habitat heterogeneity? (2) If so, how does that density dependence change along a successional gradient?

## Methods

### Study sites and data collection

The study was carried out in a typical, temperate mixed broadleaf–conifer forest in Jilin Province, northeastern China, in an experimental forest (43°51′–44°05′N, 127°35′–127°51′E) under the jurisdiction of the Jiaohe Administrative Bureau^30^. The average annual temperature is 3.8 °C. The hottest month is July with an average daytime temperature of 21.7 °C. The coldest month is January with an average day temperature of −18.6 °C. The average annual precipitation is 695.9 mm. The soil is a brown forest soil, between 20 and 100 cm deep^31^.

The analysis is based on measurements obtained from three large observational field studies. These studies, established in the summer of 2010, represent three different successional forest stages: an early successional stage (referred to as HF, 520 × 420 m), an intermediate successional stage (referred to as MF, 840 × 500 m) and a late successional stage (referred to as OGF, 500 × 600 m)^32^. In each of these large observational studies, all trees with a diameter at breast height of 1 cm or more were identified, measured and mapped.

The HF and MF areas were situated in a secondary broadleaf–conifer forest in the primary and middle stages of succession, respectively^32^. The HF study area was clear-cut about 60 years ago according to the logging records of the local forestry department. The elevation ranges from 468 to 519 m above sea level. The MF area was heavily disturbed by forest management about 60 years ago; most canopy trees are now about 100–120 years old^31^, and the elevation ranges from 459 to 517 m above sea level. In contrast, the OGF area is situated in a protected old-growth forest in a late stage of succession, with little human disturbance due to its remoteness from residential areas. The topography includes a valley between two slopes with elevations ranging from 576 to 784 m above sea level. For more details about the basic strategy regarding large permanent observational studies in China see Zhao *et al*.^33^ and Gadow *et al*.^34^. Detailed information about the vegetation in the large HF, MF and OGF permanent observational studies are presented by Zhang *et al*.^31^ and Yan *et al*.^32^.

All tree species were grouped based on maximum attainable height into five growth forms according to the classification criteria established by Piao *et al*.^10^ in the temperate forests of northeastern China: shrubs (S, ≤ 5 m in height), small understory species (US, 5 to ≤ 10 m), large understory species (UL, 10 to ≤ 20 m), small canopy species (CS, 20 to ≤ 25 m) and large canopy species (CL, > 25 m) (Table 1). Each growth form was divided into three dbh classes to define three life history stages: sapling, juvenile and adult (Table 1). In order to obtain sufficient spatial data for adequate statistical analysis, the spatial pattern of common species represented by at least 40 individual trees at each life history stage were analyzed. Accordingly, 16, 21 and 21 of these most abundant woody species with dbh ≥ 1 cm were selected in the HF, MF and OGF areas, respectively (see Supplementary Table S1).

Classification criteria based on Piao *et al*.^10^, where: S = shrubs. US = small understory tree species, UL = large understory tree species, CS = small canopy tree species and CL = large canopy species.

### Point pattern analyses

The pair correlation function g(r) was used to analyze the spatial patterns of the fully mapped tree locations at different scales. The univariate pair-correlation function g(r), which is related to the derivative of the widely used K-function^35^, is calculated as:


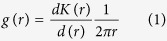


where K(r) is the expected number of points within the entire circle of a given radius r around a typical point of the pattern divided by the intensity λ of the pattern^36^,^37^. At larger scales, the values of K(r) contain the values of K(r) at smaller scales^38^,^39^, so that it confounds the effect at large distances with the effect of short distances (i.e., a cumulative effect). An important difference between the g(r) and K(r) functions is that the pair-correlation function g(r) with a circular ring replaces the entire circle of the K function, hence the g(r) function is non-cumulative and uses only points separated by the distance *r*^2^. Consequently, due to its non-cumulative property, g(r) is recommended for exploratory data analysis to identify specific scales of deviation from the null model and is considered more powerful in detecting spatial patterns across scales^38^,^40^.

### Analysis 1: Detection of environmental heterogeneity

Because large-scale environmental heterogeneity may obscure underlying density-dependent processes, we first had to assess whether the environmental conditions in each study area were heterogeneous. In 2008, Getzin *et al*.^26^ proposed a simple and effective method to describe large-scale environmental heterogeneity. They assumed that mature trees experience excessive self-thinning due to environmental filtering and are expected to have exploited all available sites, so that the combined spatial pattern of large adult trees of all species should capture strong habitat factors common to all species^26^. According to Stoyan and Penttinen^24^, the tree–tree interactions and correlations are independent beyond the scales of 10 m due to environmental heterogeneity, usually causing a large-scale aggregation pattern^2^.

Therefore, we conducted a joined pattern of all adult trees of five different growth forms separately in each study area, using a cumulative L-function with the complete spatial randomness (CSR) null model. The cumulative L-function is calculated as:


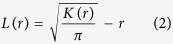


If the detected point patterns showed an additional pattern with significant deviation from the CSR model at scales > 10 m, we could infer that environmental heterogeneity exists in our study area^26^,^41^. In order to account for the effects of individual species’ identity on the detection of negative density dependence^9^,^17^, we also analyzed the spatial pattern of adults of each species individually in each plot.

### Analysis 2: Spatial population patterns

In order to determine the spatial patterns of populations of common species after removing the effects of large-scale environmental heterogeneity and further to explain whether the occurrence of density dependence is related to conspecific aggregation^9^, we used a heterogeneous Poisson process (HPP)^2^ as the null model. This method is based on the assumption of separation of scales^2^; that is to say, large-scale effects are attributed to environmental heterogeneity, typically along gradients related to topography, whereas second-order direct plant-plant interactions are affected by only small-scale effects^9^,^42^. Consequently, a heterogeneous Poisson process allows us to study typical scales at which local point-point interactions occur and to reveal the second-order characteristics of the spatial patterns of tree species by conditioning on large scale patterns^9^.

In a homogeneous Poisson process, the probability of finding any points in an area follows a Poisson distribution; in other words, any point of the pattern has an equal probability of occurring at any position in the study region^38^. On the other hand, a heterogeneous Poisson process with a function λ (x, y) varies with location (x, y) instead of with the constant intensity λ of a homogeneous Poisson process^36^,^38^. We first constructed the intensity function λ (x, y) of a given point pattern by summing all stems within a moving circular window of bandwidth R and then weighting them with a non-parametric kernel according to their distance d from the focal location (x, y)^2^,^38^. The non-parametric kernel is defined as:


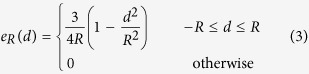


where d is the distance from a focal point and R the bandwidth. We selected a bandwidth of R = 30 m and then generated random point patterns in our study area but kept the intensity function λ (x, y) of these random points within our plots, constructed in the first step, unchanged^9^.

Significant departure from the null model was evaluated using the 5th-lowest and 5th-highest value of 999 Monte Carlo simulations of the null model to generate approximately 99% simulation envelopes^9^,^38^. Because we performed simulation envelope tests at several different spatial scales *r* simultaneously, there is chance that a Type I error may occur, i.e., the null model may be rejected even if it is true^39^. We therefore combined the common simulation envelope method with a goodness-of-fit test (GOF test)^42^ to assess significant departures from the null model^9^. We then used the data sets with an observed *p*-value < 0.005 and a rank > 995 for further analysis^2^,^39^.

### Analysis 3: Prevalence of density dependence

The extent of clustering of trees is expected to decline with increasing size class due to the effect of density-dependent self-thinning^26^,^43^. Therefore, we estimated conspecific density-dependent self-thinning by comparing the spatial patterns of two different life-history stages (i.e., smaller size class trees and adult trees) applying the g function to the null model of random labelling within a case–control design^26^,^38^,^44^.

We used the adults as the control (pattern 1) under the hypothesis that this pattern of adult trees reflects, as well as accounts for the underlying heterogeneity^26^ and smaller size class trees (i.e., saplings and juveniles) as cases (pattern 2).

The cases are a random subset of the joined pattern of cases and controls (i.e., under the random-labelling null model) if the cases do not show any additional pattern to that of the control. Therefore, under random labelling, the g-function would be: g_21_(r) = g_22_(r). We then developed the test statistic g_21_(r)–g_22_(r) to determine whether there is additional clustering within pattern 2 (saplings and juveniles) that is independent from the location of pattern 1 (adults), irrespective of whether heterogeneity is present or not^19^,^45^. If g_21_(r)–g_22_(r) < 0, we can infer that the cases show additional aggregation relative to the control pattern.

According to Getzin *et al*.^26^, the extent of the additional aggregation to decline with increasing size classes reflects density-dependent self-thinning and the strength of this decline relative to the control pattern reflects the strength of density-dependent self-thinning. As a consequence, we constructed the following function to access the change in additional aggregation from sapling to juvenile stages^9^:


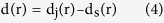


where, in the case of juveniles:


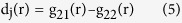


and in the case of saplings:


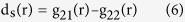


If, for a particular species, d_s_(r) < 0 and d(r) > 0, we infer that conspecific density-dependent self-thinning takes place from sapling to juvenile stages^10^. The maximum strength of density-dependent self-thinning is d_max_ and r_max_ the scale when d(r) takes the maximal value at a scale between 0–30 m. The r_thin_ reflects the scale at which density-dependent self-thinning takes place for a particular species.

In order to assess the importance of controlling for habitat preference in the analysis of density dependence, we also randomized the location of adult trees for each species and used them as pattern 1 to replace the real adult pattern which controls habitat preference and repeated the analysis described earlier^10^.

**Figure 1:**
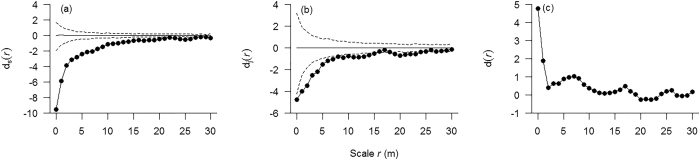
Conspecific density-dependent analysis of *Pinus koraiensis* in the MF area. *Note:* **(a**) d_s_(r) = g_21_(r)–g_22_(r) over scale *r* with saplings as cases, (**b**) d_j_(r) = g_21_(r)–g_22_(r) over scale *r* with juveniles as cases and (**c**) d(r) = d_j_(r)–d_s_(r) refers to the decline of additional aggregation from the sapling to the juvenile stage. If d_s_(r) < 0 and d(r) > 0, the conspecific density-dependent thinning takes place from sapling to the juvenile stage at scale *r*. The maximum strength of conspecific thinning (d_max_) occurred at the 0 m scale (i.e., in a 1 × 1 m grid cell).

We present the density dependence of *Pinus koraiensis*, a dominant coniferous tree species in the MF area, to illustrate the method used in this analysis (Fig. 1).

### Analysis 4: Species characteristics, successional stages and density dependence

It was necessary to determine whether the strength of the negative density dependence (measured by the maximum density dependence, d_max_) changes with the successional stage and a species characteristic such as maximal strength of conspecific aggregation (g_max_, the maximal value g(r) at a given scale) or whether that strength is simply due to species abundance. Thus, a two-way ANOVA was applied to investigate significant differences in the strength of density-dependent thinning among different species and successional stages. In this particular analysis, we only used the observations of species which were present in all three study areas and which exhibited density dependence. In addition, the nonparametric Spearman rank test was used to analyze the relationships between maximum strength of conspecific aggregation and maximum strength of density dependence as well as between species abundance and the maximum strength of density dependence.

All point pattern analyses were carried out using the R software R 3.2.2 (R Development Core Team)^46^ and the grid-based software Programita^47^.

## Results

### Analysis 1: Detection of environmental heterogeneity

**Figure 2:**
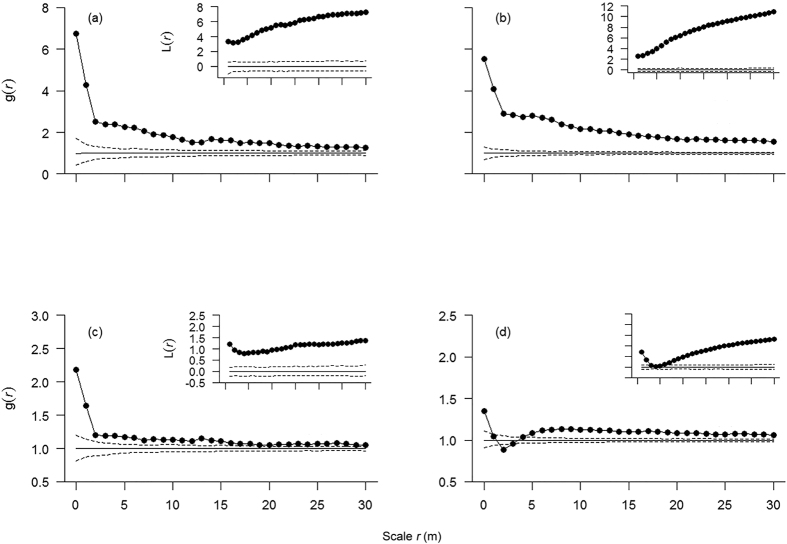
Analysis of the joint pattern of all adult trees in the HF area. *Note:* (**a**) adult trees of small understory species (US) with dbh > 6.0 cm; (**b**) adult trees of large understory species (UL) with dbh > 8.0 cm; (**c**) adult trees of small canopy species (CS) with a dbh > 12.0 cm and (**d**) adult trees of large canopy species (CL) with dbh > 15.0 cm, using the homogeneous L-function (inset figure) and the homogeneous pair-correlation function g(r) with the null model of complete spatial randomness (CSR). The 99% simulation envelopes (dashed lines) were constructed from the 5th-lowest and 5th-highest values of 999 Monte Carlo simulations of a null model of complete spatial randomness (CSR). Similar analyses in the MF and OGF areas are presented in Supplementary Fig. S1.

There were not enough common species with at least 40 individuals at each life history stage of the shrub growth form in the HF study area. Therefore, the joint patterns of all adults for shrubs and the spatial pattern of adults of each shrub species individually in HF were ignored. We compared the joint patterns of all adults for shrubs (except for HF), small understory species, large understory species, small canopy species and large canopy species separately with the CSR null model in the HF, MF and OGF areas to ascertain whether the three areas showed large-scale heterogeneity or not. The L-function showed a clear departure from the CSR model at scales r > 10 m for each growth form at each of the HF (without shrubs), MF and OGF areas (Fig. 2, MF and OGF see Supplementary Fig. S1). Additionally, the g-function was also > 1 for large-scales for each growth form at the each of the three study areas (Fig. 2, MF and OGF see Supplementary Fig. S1). We also analyzed the spatial pattern of adults of each species individually in each study area. The L-function showed that adults of all 16, 21 and 21 tested species in HF, MF and OGF showed aggregation at greater scales up to 30 m (see Supplementary Table S2). These findings indicate that each of the three study areas show large-scale heterogeneity. Thus, it became necessary to account for the effects of habitat heterogeneity when testing for density dependence.

### Analysis 2: Spatial population patterns

Except for *Maackia amurensis* in MF, all 16 and 21 tested species in HF and OGF showed a significant departure from the heterogeneous Poisson null model (i.e., the rank of the GOF test was > 995). The spatial pattern of *Maackia amurensis* in MF followed the heterogeneous Poisson null model at scales 0–30 m (see Supplementary Table S2).

**Figure 3:**
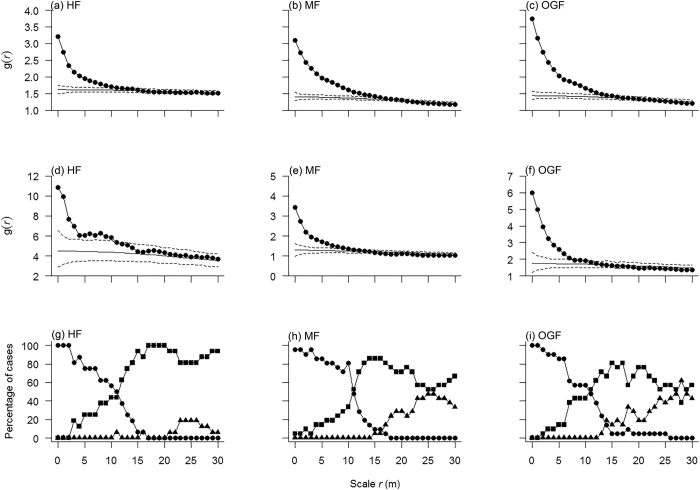
Analysis of spatial population patterns. *Note:* (**a–c**) *Acer mandshuricum*, an example of spatial population patterns of broadleaved tree species and (**d–f**) *Pinus koraiensis*, an example of spatial population pattern in a coniferous tree species of the HF, MF and OGF areas in a temperate mixed broadleaf–conifer forest in Jilin province, northeastern China. The solid circles denote the pair-correlation functions of the observed data over scale *r*, the light solid line denote the expected g function under the heterogeneous Poison null model. The 99% simulation envelopes (dashed lines) were created by calculating for each distance *r* the 5th lowest and 5th highest values from 999 Monte Carlo simulations of the null model. The null model used the non-parametric kernel estimate of the intensity function λ (x, y) of the pattern with a bandwidth of R = 30 m. The ring width for estimation of the pair-correlation function was 3 m, with a cell size of 1 × 1 m. (**g–i**) Proportion of species showing significant aggregation (solid circles), regularity (solid triangles) and randomness (solid squares) over different scales in the HF, MF and OGF areas, respectively.

In order to assess the scale-dependent effects of the second-order characteristics of the population patterns, we calculated for each detail scale *r* the percentage of species for which the pair-correlation function was outside the simulation envelopes (i.e., above or below the 5th highest or 5th lowest value of the pair-correlation function in the 999 Monte Carlo simulations). The percentage of aggregations decreased sharply and had a clearly similar directional trend with increasing spatial scales in HF, MF and OGF (Fig. 3g–i). Specifically, in the HF area, 13 of the 16 species (81.3%) were aggregated at scales from 0 to 4 m, nine species (56.3%) at scales from 5 to 10 m and only one species (6.3%) at scales from 11 to 16 m, while not a single species showed aggregation beyond the 17 m scale (Fig. 3g). In MF, 17 of the 21 species (81.0%) were aggregated at scales from 0 to 4 m, 14 species (66.7%) at scales from 5 to 10 m and only two species (9.5%) were aggregated at scales from 11 to 16 m. Again, no species showed aggregation beyond the 18 m scale (Fig. 3h). In the OGF area, 18 of the 21 species (85.7%) were aggregated at scales from 0 to 4 m, 12 species (57.1%) at scales from 5 to 10 m, and only one species (4.8%) aggregated at scales from 11 to 16 m. Except for *Carpinus cordata* (which showed aggregation up to the 25 m scale), no species showed aggregation beyond the 15 m scale (Fig. 3i). In contrast, the percentage of regular pattern increased with scales from 21, 15 and 13 m to 30 m for the HF, MF and OGF areas, respectively (Fig. 3g–i).

### Analysis 3: Prevalence of density dependence

**Figure 4:**
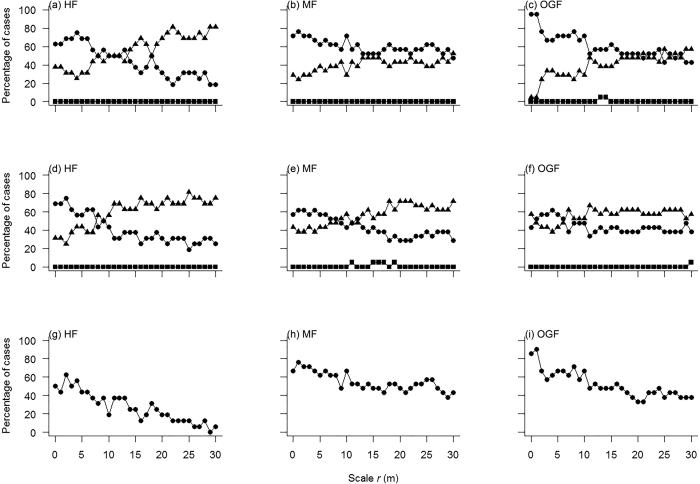
Analysis of conspecific density-dependent self-thinning as a function of scale in three areas. *Note:* (**a**–**f**) Proportion of species showing the test statistic g_21_(r)–g_22_(r) < 0 (solid circles), g_21_(r)–g_22_(r) > 0 (solid squares) and g_21_(r)–g_22_(r) = 0 (solid triangles) over scales at the HF, MF and OGF areas, (**a–c**) saplings as cases and (**d–f**) juveniles as cases. (**g–i**) Proportion of examined species showing density dependence at detailed scales at the HF, MF and OGF areas.

In the HF observational study, for saplings, 12 of the 16 species tested exhibited additional aggregation patterns when compared with adults, three species showed random patterns and no species showed any regular patterns. For juveniles, 13 of the 16 species exhibited additional clustering relative to adults, three species showed random patterns and again not a single species showed a regular pattern. In MF, for saplings, 18 of the 21species tested exhibited additional aggregation patterns relative to adults, three species showed random patterns and no species showed any regular pattern. For juveniles, 17 of the 21 tested species exhibited additional clustering relative to adults, three species showed random patterns and two species showed regular patterns. In OGF, for saplings, all 21 species exhibited additional aggregation patterns relative to adults; no species showed a random pattern, nor did any species show a regular pattern. For juveniles, however, only 16 out of 21 species tested exhibited additional clustering relative to adults, four species showed random patterns and one species showed a regular pattern (see Supplementary Table S2). Overall, the percentage of species showing additional clustering relative to adults, decreased from saplings to juveniles over all examined scales in the MF and OGF area, but this tendency did not apply to the HF area (Fig. 4a–f, see Supplementary Table S2).

The proportion of examined species showing density dependence exhibited significant differences among the three successional stages (ANOVA; F = 36.74, *p* < 0.000). Specifically, the proportion showing density dependence in OGF accounted for the greatest percentage, followed by MF and HF. In HF, 11(68.8%) out of 16 species, in MF 18(85.7%) out of 21 and in OGF all 21 species showed a decline of strength of additional clustering from saplings to juveniles (see Supplementary Table S2), which suggests that most species show negative density dependence across these temperate forests. Our analysis also showed that the prevalence for density dependence increases across successional stages.

There was a clear tendency for the percentage of cases showing density dependence to decrease with increasing spatial scale *r* (Fig. 4g–i) in all three study areas. For all species examined, the maximum strength of density dependence predominantly occurred at scales smaller than 10 m, except for *Carpinus cordata* in HF. 8, 13 and 18 species reached the maximum strength of density dependence at the 0 m scale (in a 1 × 1 m grid cell) in HF, MF and OGF respectively (see Supplementary Table S2). These results show that, as expected, negative density dependence occurs at very close distances among immediate neighbors.

### Analysis 4: Species characteristics, successional stages and density dependence

The strength of density-dependent self-thinning is closely related with species abundance and maximum strength of conspecific aggregation g_max_. Specifically, the strength of density-dependent self-thinning as measured by d_max_ decreases with increasing species abundance (Spearman’s rho = −0.60, S = 33222, *P* = 5.12 e–6), suggesting that rare species are more vulnerable to the effect of density dependence than the more common species. The strength of density-dependent self-thinning, however, was positively correlated with the maximum strength of conspecific aggregation (Spearman’s rho = 0.80, S = 4588.6, *P* = 2.54 e–11).

A two-way ANOVA showed that the strength of density-dependent self-thinning significantly varied among species (F = 6.91, *P* = 0.005). In contrast, the strength of density-dependent self-thinning was not significantly different among the three different successional stages (F = 0.62, *P* = 0.55). On the whole, the strength of density-dependent self-thinning seems significantly affected by the characteristics of different species, rather than by the successional stage.

In contrast, the prevalence of density dependence was different among the three successional stages and showed increases along the successional gradient (68.8%, 85.7% and 100.0% in HF, MF and OGF, respectively). According to Bruelheide *et al*.^29^, the effect of negative density might accumulate and competition for limited resources and spaces may become more intense over time. Our results support the assumption of a cumulative effect of density dependence, which is further supported by the prevalence of density-dependent self-thinning with and without factoring out the effect of habitat heterogeneity. Regardless of whether habitat heterogeneity was excluded or not, only in the OGF area did all species exhibit negative density dependence.

## Discussion

In line with previous studies, we could demonstrate that environmental heterogeneity may introduce a bias in the detection of density dependence. It was therefore important to eliminate the effect of habitat heterogeneity when testing for density dependence in the northeastern temperate forest. However, in all three study areas, the percentage of cases showing density dependence at greater scales (*r* ≥ 15 m), without factoring out habitat heterogeneity, was significantly higher than when habitat heterogeneity was eliminated (t-test: *P* = 0.0002 in HF, 4.09e-06 in MF and 0.0002 in OGF; see Supplementary Fig. S2).

One possible explanation for this difference could be that when habitat heterogeneity was not eliminated, conspecific self-thinning tended to be detected at greater spatial scales, and was likely driven by unfavorable habitat rather than by tree–tree interactions^10^. Additionally, the number of species showing density dependence was also different with and without factoring out habitat heterogeneity in the HF and MF areas. Two of the 16 and one of the 21 species examined did not show density dependence when habitat heterogeneity was not eliminated in the HF and MF areas (see Supplementary Fig. S3). *Padus racemosa, Rhamnus davurica* and *Acer tegmentosum* did not show density dependence when habitat heterogeneity was eliminated, but density dependence emerged after eliminating habitat heterogeneity in HF. *Juglans mandshurica* and *Phellodendron amurense* did not show any density dependence in HF, irrespective of whether habitat heterogeneity effects were removed or not. In the MF area, only *Maackia amurensis* and *Abies holophylla* did not display density dependence after controlling for habitat heterogeneity while *Juglans mandshurica* did not show density dependence before and after controlling for habitat heterogeneity. In OGF, all 21 species examined showed density dependence both with and without controlling for habitat heterogeneity.

In line with previous studies, the results were different before and after factoring out habitat heterogeneity (Fig. 4, Supplementary Fig. S3). Zhu *et al*.^9^ examined density dependence of 47 common tree species in a subtropical forest. In that study, the percentage of cases showing density dependence was higher at small scales than at greater scales after removing the effect of habitat heterogeneity. However, the percentage of cases showing density dependence was not different at small scales from that at greater scales when the effect of habitat heterogeneity was not removed. They also found that the number of species showing density dependence was different with and without factoring out habitat heterogeneity. Piao *et al*.^10^ could show that the significance and scale of density dependence were different with and without factoring out habitat heterogeneity in a temperate old-growth forest of northeast China. Thus, earlier findings combined with the results of our study suggest that the effects of habitat-induced self-thinning on detecting density-dependent self-thinning should be taken into account.

The results of this study provides clear evidence of density-dependent self-thinning. 68.8 percent of the 16 investigated species, in HF, 85.7 percent of the 21 investigated species in MF and all the investigated 21 species in OGF showed density dependent effects for individual trees with a dbh greater or equal to 1 cm. These proportions are consistent with most other studies on density dependence at later life stages in tropical forests^7^,^11^, subtropical forests^9^,^17^, as well as temperate forests^10^. Hence, there is no clear evidence of any significant difference between tropical forests and other zonal forests in terms of the generality of density dependent self-thinning. We may thus assume that negative density dependence, - a mechanism which explains diversity in species coexistence, is not exclusive to tropical forests but is also encountered in other zonal forests^48^,^49^.

It is well known that the transition from the seedling to the sapling stage has been regarded as a bottleneck in individual tree establishment^50^, because individuals at the early life-stages are more susceptible to both biotic and abiotic constraints^1^. Therefore, many studies have focused on detecting the prevalence of density dependence in the very early life-stages. As a consequence, these studies have provided strong evidence for the important role of negative density dependence at the seedling stage^7^,^11^,^13^,^32^,^51^,^52^,^53^,^54^. Interestingly, relatively few studies of density dependence have been conducted involving later life-history stages in temperate forest communities. In this study, we could demonstrate that density dependence is also prevalent at later life stages.

Most species reached a maximum degree of density dependent self-thinning (d_max_) at the 0 m scale (in a 1 × 1 m grid cell), while the percentage of cases showing density dependence decreased with increasing spatial scale *r*. This suggests that, as expected, density-dependent self-thinning occurs at very close distances among neighbors. In contrast, only one species (*Carpinus cordata*) in the HF area reached the d_max_ at scales > 23 m, indicating that in this particular case, the effect could have been caused by habitat heterogeneity rather than tree–tree interactions.

The multi-facetted relationships between species abundance and density dependence remains an issue subject to debate in the field of community ecology^49^. According to Murrell^27^, density-dependent effects and their resulting patterns may be governed by species abundance as well as by individual species properties. Clear evidence emerged in this study that the d_max_ decreases with species abundance, suggesting that rare species are more vulnerable to the effect of density dependence than more common species. This finding is consistent with the results of Zhu *et al*.^8^ and Guo *et al*.^17^. In species showing density dependence, the mean values of d_max_ (11.2 in HF, 8.1 in MF and 15.3 in OGF) for abundant individuals (n > 1000) were much lower than those of less abundant individuals (n < 1000; 25.2 in HF, 36.2 in MF and 62.2 in OGF). These results are consistent with the view that rare species suffer more from density dependence than common species^8^. However, the proportion of abundant species showing density dependence (87.5% in HF and 91.7% in MF) was higher than that of less abundant species (50% in HF and 77.8% in MF). This result suggests that density dependence tends to affect primarily the more abundant species. Except for *Juglans mandshurica*, the species not subject to density-dependent self-thinning are the less abundant ones in HF and MF. Although *Juglans mandshurica* is an abundant species in the HF and MF study areas, this species has not shown the effect of density dependence. A possible reason is that the number of mature *Juglans mandshurica* trees is considerably greater than that of *Juglans mandshurica* saplings or juveniles in HF and MF (see Supplementary Table S1). Consequently, our result confirms a previous conclusion that large trees are less vulnerable to herbivores and pathogens than smaller ones^8^,^15^,^50^. We also found that the strength of density-dependent self-thinning significantly varied among species (*P* = 0.005).

Moreover, we found that the strength of density-dependent self-thinning is positively related with the maximum strength of conspecific aggregation (g_max_). For example, the less abundant species *Larix gmelinii* (n = 184), *Acer tegmentosum* (n = 564) and *Cerasus maximowiczii* (n = 293) had the highest g_max_ values (81.7, 44.5 and 83.2, respectively) and simultaneously the highest d_max_ values (87.3, 66.0 and 164.6) of all species examined in HF, MF and OGF, respectively. In contrast, *Juglans mandshurica* being an abundant species in HF and MF (n = 4568 and 2116, respectively) had much lower g_max_ values (1.9 in HF and 2.6 in MF) than the mean g_max_ values (22.1 and 17.3) of all species examined. This result could provide another explanation why the abundance of *Juglans mandshurica* does not show density dependence in either HF or MF.

A two-way ANOVA showed that the strength of density-dependent self-thinning is significantly affected by the specific characteristics of the species, rather than by successional stage. Species variation in density dependence may be caused by differences in morphological, physiological and allocation traits^17^. For example, shade-tolerant species can adapt to reduced radiation under canopy and may experience less density-dependent mortality than shade-intolerant species^55^. On the other hand, density-dependent self-thinning was not significantly affected by successional stage. This may be due to the fact that the successional differences between the three study areas were not sufficiently great to expose such differences.

In conclusion, this study provides overwhelming evidence for the prevalence of density dependence after controlling the confounding effect of habitat heterogeneity in the study area. Our findings demonstrate that it is crucial to control for the effect of habitat heterogeneity in studies of density dependence in plant communities. Our findings reflect that the prevalence of density dependence is different among three successional stages. We also provide evidence that the strength of density dependence is closely related with species abundance and maximum strength of conspecific aggregation.

## Additional Information

**How to cite this article**: Yao, J. *et al*. Effects of density dependence in a temperate forest in northeastern China. *Sci. Rep.* **6**, 32844; doi: 10.1038/srep32844 (2016).

## Supplementary Material

Supplementary Information

## Figures and Tables

**Table 1:** Life stage classifications based on dbh (cm) for trees of different growth forms in a temperate forest in northeastern China.

Life-stage	S	US	UL	CS	CL
Saplings	1.0–1.5	1.0–3.0	1.0–5.0	1.0–6.0	1.0–8.0
Juveniles	1.6–2.0	3.1–6.0	5.1–8.0	6.1–12.0	8.1–15.0
Adults	>2.0	>6.0	>8.0	>12.0	>15.0
