# Supplementary material for: Effects of density dependence in a temperate forest in northeastern China
Source: Sci Rep. 2016 Sep 8;6:32844. doi: 10.1038/srep32844 (PMC5015110; doi:10.1038/srep32844)
Supplement: Supplementary Information [file srep32844-s1.pdf]

Supplementary Informations

Title: Effects of Density Dependence in a Temperate Forest in Northeastern China

Authors: Jie Yao[1], Xinna Zhang[1], Chunyu Zhang[1]*, Xiuhai Zhao[1] & Klaus von Gadow[2]

[1] Key Laboratory for Forest Resources & Ecosystem Processes of Beijing, Beijing Forestry University, No. 35 Qinghua East Road, Haidian District, Beijing 100083, China.

[2] Faculty of Forestry and Forest Ecology, Georg-August-University Göttingen, Büsgenweg 5, D-37077 Göttingen, Germany; Department of Forest and Wood Science, University of Stellenbosch, South Africa

* Corresponding author

Telephone number: +8601062336189. E-mail addresses: zcy_0520@163.com (C. Zhang)

Supplementary Table S1 List of species examined and number of individual trees of different size classes for each species in the HF, MF and OGF areas

Study areas	Species	Family	Growth forms	Number of individuals			
				Adults	Juveniles	Saplings	Total
HF	*Acer mandshuricum*	*Aceraceae*	CS	614	998	3732	5344
	Acer mono	*Aceraceae*	CS	595	1166	2804	4565
	Acer tegmentosum	*Aceraceae*	UL	70	44	47	161
	Carpinus cordata	*Betulaceae*	UL	148	148	297	593
	Fraxinus mandschurica	*Oleaceae*	CL	1014	568	330	1912
	Juglans mandshurica	*Juglandaceae*	CL	3489	861	218	4568
	Larix gmelinii	*Pinaceae*	CL	57	87	40	184
	Padus racemosa	*Rosaceae*	US	108	133	76	317
	Phellodendron amurense	*Rutaceae*	CS	94	81	77	252
	Pinus koraiensis	*Pinaceae*	CL	340	148	154	642
	Rhamnus davurica	*Rhamnaceae*	UL	59	121	55	235
	Syringa reticulate var. *amurensis*	*Oleaceae*	US	683	388	79	1150
	Tilia amurensis	*Tiliaceae*	CL	228	195	222	645
	Ulmus davidiana var. *japonica*	*Ulmaceae*	CS	885	1246	1352	3483
	Ulmus laciniata	*Ulmaceae*	CS	306	645	918	1869
	Ulmus macrocarpa	*Ulmaceae*	UL	1369	706	646	2721
MF	*Abies holophylla*	*Pinaceae*	CL	270	107	134	511

	Acer barbinerve	*Aceraceae*	S	1530	887	667	3084
	Acer mandshuricum	*Aceraceae*	CS	1422	1083	4440	6945
	Acer mono	*Aceraceae*	CS	2780	1712	5303	9795
	Acer tegmentosum	*Aceraceae*	UL	249	143	172	564
	Acer triflorum	*Aceraceae*	CS	374	202	358	934
	Carpinus cordata	*Betulaceae*	UL	3306	2316	4405	10027
	Corylus mandshurica	*Betulaceae*	S	635	447	376	1458
	Fraxinus mandschurica	*Oleaceae*	CL	1638	453	100	2191
	Juglans mandshurica	*Juglandaceae*	CL	1990	82	44	2116
	Maackia amurensis	*Leguminosae*	UL	111	44	46	201
	Padus racemosa	*Rosaceae*	US	167	292	241	700
	Pinus koraiensis	*Pinaceae*	CL	1394	397	762	2553
	Quercus mongolica	*Fagaceae*	CL	517	179	156	852
	Sorbus alnifolia	*Rosaceae*	CS	213	222	269	704
	Syringa reticulate var. *amurensis*	*Oleaceae*	US	454	1589	894	2937
	Tilia amurensis	*Tiliaceae*	CL	1570	649	569	2788
	Tilia mandshurica	*Tiliaceae*	CS	423	130	72	625
	Ulmus davidiana var. *japonica*	*Ulmaceae*	CS	579	501	774	1854
	Ulmus laciniata	*Ulmaceae*	CS	571	446	1222	2239
	Ulmus macrocarpa	*Ulmaceae*	UL	247	55	108	410
OGF	*Abies holophylla*	*Pinaceae*	CL	210	104	383	697
	Acer barbinerve	*Aceraceae*	S	5455	1180	302	6937
	Acer mandshuricum	*Aceraceae*	CS	933	1012	3502	5447

Acer mono	*Aceraceae*	CS	1334	927	1851	4112
Acer tegmentosum	*Aceraceae*	UL	994	428	396	1818
Acer ukurunduense	*Aceraceae*	US	808	810	440	2058
Betula costata	*Betulaceae*	CS	620	155	54	829
Carpinus cordata	*Betulaceae*	UL	2605	1468	2609	6682
Cerasus maximowiczii	*Rosaceae*	UL	96	52	145	293
Corylus mandshurica	*Betulaceae*	S	3583	1491	313	5387
Euonymus rehderianus	*Celastraceae*	S	577	218	103	898
Euonymus verrucosus	*Celastraceae*	S	133	168	117	418
Fraxinus mandschurica	*Oleaceae*	CL	235	96	77	408
Juglans mandshurica	*Juglandaceae*	CL	711	78	45	834
Padus racemosa	*Rosaceae*	US	163	312	247	722
Pinus koraiensis	*Pinaceae*	CL	448	141	750	1339
Sorbus alnifolia	*Rosaceae*	CS	391	297	377	1065
Syringa reticulate var. *amurensis*	*Oleaceae*	US	818	1382	825	3025
Tilia amurensis	*Tiliaceae*	CL	1172	445	345	1962
Ulmus laciniata	*Ulmaceae*	CS	1405	586	822	2813
Ulmus macrocarpa	*Ulmaceae*	UL	115	47	51	213

Note: Growth forms, S, US, UL, CS and CL denote shrubs, small understory tree species, large understory tree species, small canopy tree species and large canopy tree species.

Supplementary Figure S1 Analysis of the joint pattern of all adult trees in MF and OGF

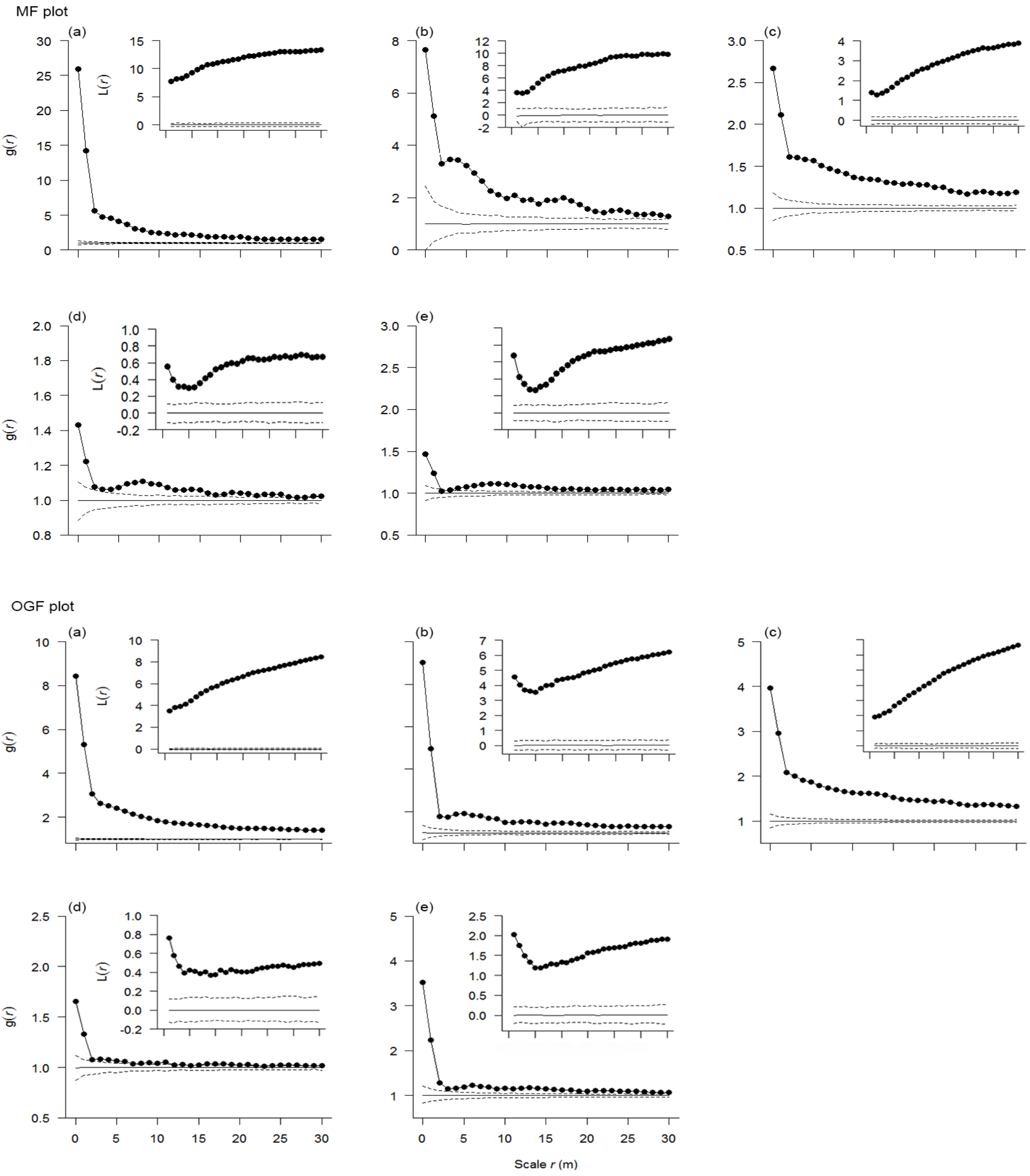

Note: Patterns of all adult trees in MF and OGF with (a) adult trees of shrubs (S) with dbh > 2.0 cm; (b) adult trees of small understory species (US) with dbh > 6.0 cm (c) adult trees of large understory species (UL) with a dbh > 8.0 cm; (d) adult trees of small canopy species (CS) with dbh > 12.0 cm, (e) adult trees of large canopy species (CL) with dbh > 15.0 cm, using the homogeneous L-function (inset figure) and the homogeneous pair-correlation function g(r) with the null model of complete spatial randomness (CSR). The 99% simulation envelopes (dashed lines) were constructed from the 5th-lowest and 5th-highest values of 999 Monte Carlo simulations of a null model of complete spatial randomness (CSR).

Supplementary Table S2 Analyses of habitat heterogeneity, spatial population patterns and conspecific density dependence in HF, MF and OGF

Study areas	Species	Habitat heterogeneity (CSR)		Spatial population pattern (HPP)	Conspecific density dependence (RL)				
		g(r)	L(r)		r_s (m)	r_j (m)	r_{thin} (m)	r_{max} (m)	d_{max}
HF	*Acer mandshuricum*	0–30	1–30	0–11	0–30	0–30	0–2, 7–9, 13–15	0	0.9
	Acer mono	0–30	1–30	0–12, 16(–), 21–28(–)	0–16	0–3, 7–10, 13–15, 18, 29–30	2–6, 11–12	2	0.5
	Acer tegmentosum	0–28	1–30	0–7, 11–13	0–30(r)	0–30(r)	None	None	None
	Carpinus cordata	0–30	1–30	0–9, 26(–)	0–14, 17–30	0–11, 19, 26–29	11–12, 18, 21–25, 28, 30	23	1.4
	Fraxinus mandschurica	0–30	1–30	0–10	0–28	0–24, 28	0–28	0	5.2
	Juglans mandshurica	0–1, 3–30	1–30	0–1, 4–12	4–6, 9–10, 18, 23–24	0–30	None	None	None
	Larix gmelinii	0–30	1–30	0–16, 29(–)	2–4, 11–13	2	2–4, 11–13	3	87.3
	Padus racemosa	0–20, 27–30	1–30	0–4, 23–24(–), 27–28(–)	0–30(r)	0–30(r)	None	None	None
	Phellodendron amurense	0–1, 27–28	1–30	0–2, 24(–)	0–30(r)	2, 6–7, 9	None	None	None
	Pinus koraiensis	0–30	1–30	0–10	0–18, 24–30	0–30	0, 2, 4, 9	0	3.6
	Rhamnus davurica	0–1	1–30	0–2, 23(–)	0–30(r)	0–30(r)	None	None	None
	Syringa reticulate var. *amurensis*	0–30	1–30	0–7, 11(–), 25(–)	0–9, 18–21	0–7, 12–15, 17, 19–20	0–9, 18–21	0	60.4
	Tilia amurensis	0–30	1–30	0–4, 30(–)	0–7	0–1	0–7	0	8.3
	Ulmus davidiana var. *japonica*	0–30	1–30	0–14	0–20	0–9	0–20	0	3.3
	Ulmus laciniata	0–1, 9–10, 13–14, 16–30	1–30	0–11	0–14	0–7, 22–24	0–14	0	2.1
	Ulmus macrocarpa	0–30	1–30	0–14, 25–26(–)	0–6, 13, 15, 17–19	0–5, 10	0–6, 13, 15, 17–19	0	5.7
MF	*Abies holophylla*	0–30	1–30	0–9	0–30(r)	19(+)	None	None	None
	Acer barbinerve	0–30	1–30	0–10, 18–20(–), 22–30(–)	0–4, 6–10	0–2	0–4, 6–10	0	13.0
	Acer mandshuricum	0–30	1–30	0–16, 24–30(–)	0–30	0–19, 21–30	0–30	0	1.7
	Acer mono	0–30	1–30	0–17	0–30	0–30	0–30	2	2.0
	Acer tegmentosum	0–11, 14–24, 27–30	1–30	0–5, 18–19(–), 23–28(–)	0–10, 12, 18, 23–26	11–13	0–10, 18, 23–26	0	66.0
	Acer triflorum	0–30	1–30	0–11, 30(–)	0–30	0–9, 12, 17–26, 30	0–30	10	7.5

	Carpinus cordata	0–30	1–30	0–12, 26–30(–)	0–29	0–20, 23–24, 27–29	0–16, 22–23	0	0.8
	Corylus mandshurica	0–30	1–30	0–10, 18–30(–)	0–1, 10, 18–29	28–30	0–1, 10, 18–28	0	14.2
	Fraxinus mandschurica	0–30	1–30	0–11	2, 17–20	0–17, 21–29	2, 17–20	2	5.0
	Juglans mandshurica	0–30	1–30	0–11	0–30(r)	6, 17	None	None	None
	Maackia amurensis	0–30	1–30	0–30(r)	0–30(r)	1–5, 11(+), 15–17(+), 25–29	None	None	None
	Padus racemosa	0–19, 24, 27–28	1–30	0–10, 21–30(–)	0–30	0–16, 20, 27	0–11, 13–30	0	64.0
	Pinus koraiensis	0–30	1–30	0–10, 17–20(–), 22–30(–)	0–30	0–15, 19–25, 27	0–19, 24–26, 30	0	4.8
	Quercus mongolica	0–28	1–30	0–3, 23(–)	3–8, 10–30	4	3–8, 10–30	0	12.6
	Sorbus alnifolia	0–8, 10–12, 15–17, 22–24, 27–28	1–30	0–7, 15(–), 17(–), 19–26(–)	0–6, 10–12	0–30(r)	0–6, 10–12	0	22.1
	Syringa reticulate var. *amurensis*	0–29	1–30	0–11	0–30	0–30	0–30	0	14.7
	Tilia amurensis	0–15, 18–29	1–30	0–3, 16(–), 19–21(–), 25(–)	0–30	0–13, 25–26	0–8, 10–12, 14–30	0	20.6
	Tilia mandshurica	0–30	1–30	0–14, 22–29(–)	1	0–30(r)	1	1	54.7
	Ulmus davidiana var. *japonica*	0–30	1–30	0–13	0–30	0–8, 15–19, 28–30	0–16, 19–28	0	7.2
	Ulmus laciniata	0–10, 18–20, 22–26	1–30	0–12, 17–18(–), 23–29(–)	0–17, 21, 25–27	0–12, 14–16	0–8, 12–14, 25–27	0	5.4
	Ulmus macrocarpa	0–19, 22–25, 27–30	1–30	0–8, 20–21(–)	0–3, 29–30	0–30(r)	1–3, 29–30	2	26.4
OGF	*Abies holophylla*	0–30	1–30	0–10	0–10, 15–20, 26, 28	6, 29	0–4, 8–10, 16–19, 26	0	5.1
	Acer barbinerve	0–30	1–30	0–12, 16–30(–)	0–10, 12–13	0–30	0–10, 12–13	0	43.2
	Acer mandshuricum	0–30	1–30	0–13, 28–30(–)	0–30	0–30	0–1, 5–8, 10–18, 22–30	0	0.5
	Acer mono	0–30	1–30	0–14, 29–30(–)	0–24, 26	0–30	1–12, 16–17	2	1.2
	Acer tegmentosum	0–30	1–30	0–4, 14–15(–), 18–19(–), 24–30(–)	0–30	2	0–30	0	36.4
	Acer ukurunduense	0–30	1–30	0–2, 4–10, 12–13, 18, 22(–)	0–2, 8, 10, 15–16	30(+)	0–2, 8, 10, 15–16	0	29.5
	Betula costata	0–15, 20–21	1–30	0–6, 16–19(–), 21–24(–)	0–1, 5–11, 14–15	1–6, 8–10, 13, 15–27, 29–30	0–1, 5–11, 14–15	0	75.8
	Carpinus cordata	0–30	1–30	0–25	0–30	0–30	0–27, 29–30	0	1.6
	Cerasus maximowiczii	0–28	1–30	0–11, 18–30(–)	0–2, 22–24, 27–30	0–30(r)	0–2, 22–24, 27–30	0	164.6
	Corylus mandshurica	0–30	1–30	0–14, 20–30(–)	0–30	0–30	0–30	0	32.5
	Euonymus rehderianus	0–30	1–30	0–6, 14–19(–), 24(–), 26(–), 28–30(–)	0–5	0–6	0–5	0	35.7

Euonymus verrucosus	0–17, 20–29	1–30	0–3, 22–30(–)	1	0–30(r)	1	1	14.3
Fraxinus mandschurica	0–22, 26–28, 30	1–30	0–5, 25(–), 27–29(–)	0–1, 13–14(+)	0–30(r)	0–1	0	35.0
Juglans mandshurica	0–30	1–30	0–1, 6	0	4–5, 28–29	0	0	51.4
Padus racemosa	0–21, 23–24, 28–30	1–30	0–11, 23–30(–)	0–30	2–4, 9–10, 21–24	0–30	0	59.7
Pinus koraiensis	0–30	1–30	0–7, 28(–)	0–30	0–4, 7–30	4–6	5	1.3
Sorbus alnifolia	0–4, 9–12, 17–20, 29	1–30	0–6, 14–16(–), 18–20(–), 23–28(–)	0–13	0–30(r)	0–13	0	10.9
Syringa reticulate var. *amurensis*	0–30	1–30	0–11, 18(–), 26–28(–)	0–28	0–10, 12–16	0–28	0	8.8
Tilia amurensis	0–30	1–30	0–6, 13–14(–), 28(–)	0–1, 6–8	1, 3–6, 8	0–1, 6–8	0	14.9
Ulmus laciniata	0–4, 8–13, 15–30	1–30	0–12	0–30	0–30	0–30	0	3.0
Ulmus macrocarpa	0–30	1–30	0–12, 27(–)	0–10, 12–14, 24	5	0–10, 12–14, 24	0	118.0

Note: In analysis 1 and 2, scales at which species show significant regularity are indicated by a "–" in parenthesis, scales showing significant aggregation are not indicated by a "+" in parenthesis; only the entire range of the 0–30 m scales showing randomness is indicated by a "r". In analysis 3 for conspecific density dependence self-thinning, $d(r) = d_j(r) - d_s(r)$ for accessing the change in additional aggregation from sapling to juvenile stages, where $d_j(r) = g_{21}(r) - g_{22}(r)$ and $d_s(r) = g_{21}(r) - g_{22}(r)$ over the scales *r* where the cases are juveniles and saplings, respectively. For a particular species, if $d_s(r) < 0$ and $d(r) > 0$, then conspecific density-dependent self-thinning takes place. At the 0 – 30 m scale, d_{max} is the maximum strength of density-dependent self-thinning and r_{max} the scale when d(r) takes on the maximal value. The r_{thin} reflects the scales at which conspecific density-dependent self-thinning takes place for a particular species. If $g_{21}(r) - g_{22}(r) > 0$ is statistically significant, the scale is indicated by a "+" in parenthesis; if $g_{21}(r) - g_{22}(r) = 0$, the corresponding scale is indicated by an "*r*", but given only over the entire 0–30 m scale, not at partial scales; if $g21(r) - g22(r) < 0$ is significant, the scale is presented, but not signed by a "–" in parenthesis. "None" denotes that species do not show conspecific density dependence self-thinning at the r =0 – 30 m scale.

Supplementary Figure S2 Analysis of conspecific density dependence self-thinning with and without factoring out habitat heterogeneity over larger scales (r ≥ 15 m;) in three study areas

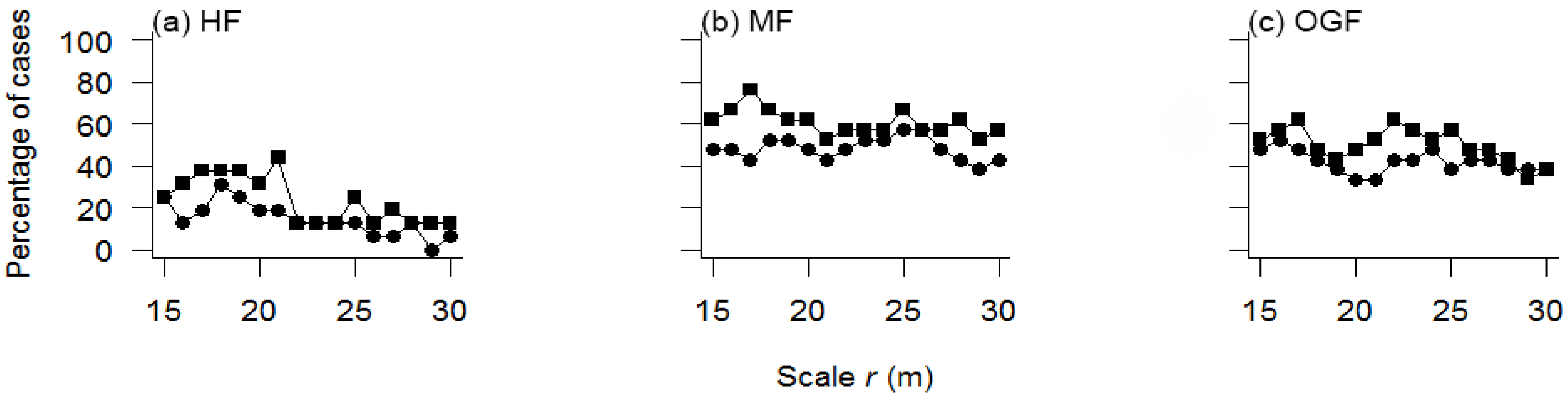

Note: Solid circles denote the percentage of cases showing conspecific density dependence self-thinning with factoring out the effects of habitat heterogeneity and solid squares denote the percentage of cases showing density dependence self-thinning without factoring out the effects of habitat heterogeneity at the larger scales, i.e., r = 15 – 30 m in the three areas.

Supplementary Figure S3 Analysis of conspecific density dependence self-thinning as a function of scale without factoring out habitat heterogeneity in three areas

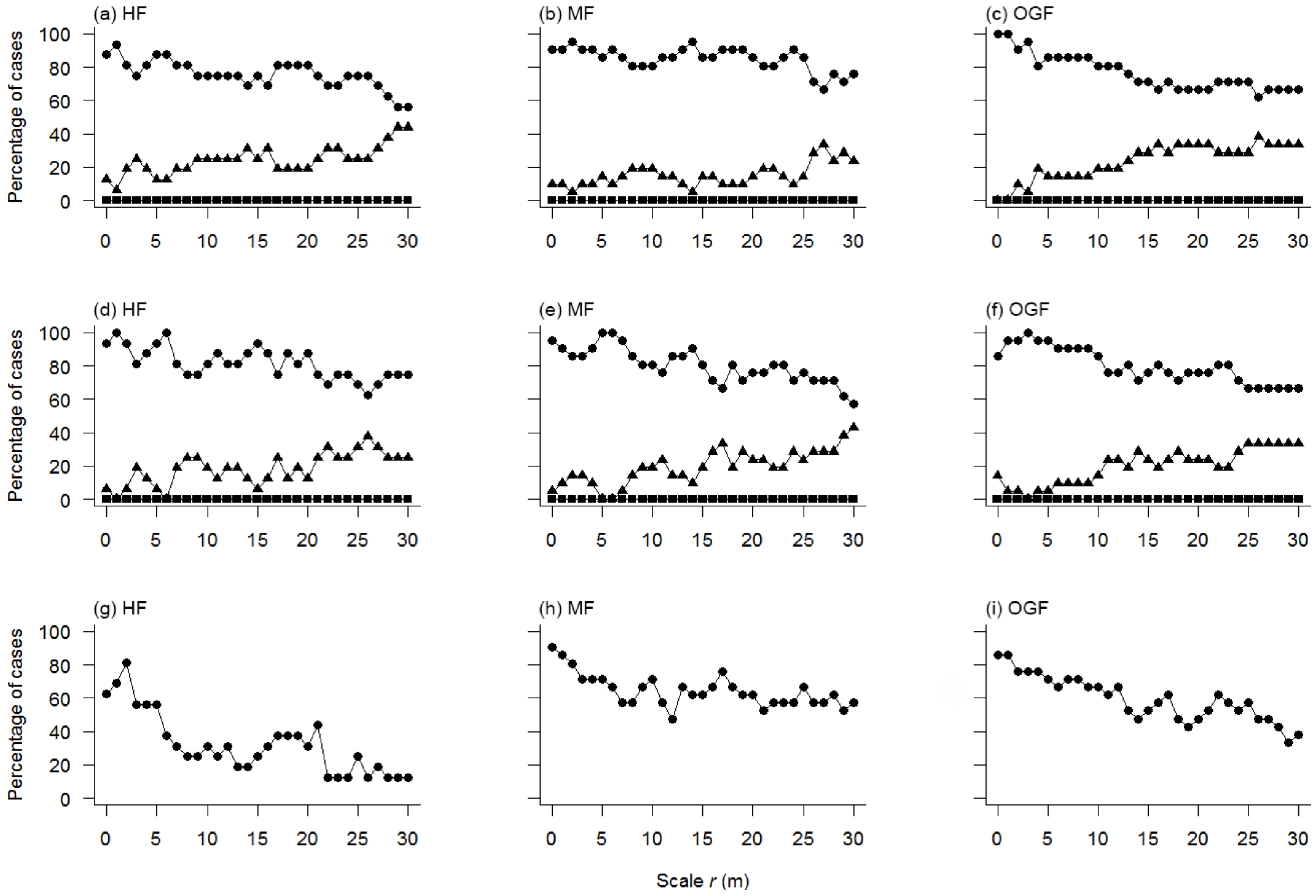

Note: In this analysis, we randomized the locations of adult trees for each species and used them as pattern 1 to replace the real adult pattern which controls habitat preference. When the cases are juveniles $d_j(r) = g_{21}(r) – g_{22}(r)$ and $d_s(r) = g_{21}(r) – g_{22}(r)$ when saplings over scales *r*. For (a – f): the proportion of species showing the test statistic $g_{21}(r) – g_{22}(r) < 0$ (solid circles), $g_{21}(r) – g_{22}(r) > 0$ (solid squares) and $g_{21}(r) – g_{22}(r) = 0$ (solid triangles) over all scales in HF, MF and OGF, (a – c) saplings as cases and (d – f) juveniles as cases. For (g – i): the proportion of species examined showing density dependence without factoring out habitat heterogeneity at detailed scales in HF, MF and OGF.
